# MCQ-Balance: a method to monitor patients with balance disorders and improve clinical interpretation of posturography

**DOI:** 10.7717/peerj.10916

**Published:** 2021-02-23

**Authors:** Juan De la Torre, Javier Marin, Marco Polo, Eva M. Gómez-Trullén, Jose J. Marin

**Affiliations:** 1 IDERGO (Research and Development in Ergonomics) Research Group, I3A (Aragon Institute of Engineering Research), Zaragoza, Spain; 2 Department of Biomedical Engineering, Universidad de Zaragoza, Zaragoza, Spain; 3 Department of Design and Manufacturing Engineering, Universidad de Zaragoza, Zaragoza, Spain; 4 MD Physical Medicine and Rehabilitation, Hospital of Alcañiz, Teruel, Spain; 5 Department of Physical Medicine and Rehabilitation and Nursing, Health Sciences Faculty, Universidad de Zaragoza, Zaragoza, Spain

**Keywords:** Balance sensory systems, Objective information, Patient-level analysis, Stabilometric platform, Monitoring, Vertigo, Personalized medicine, Methodology

## Abstract

**Background:**

An estimated 20% to 30% of the global population has suffered a vertiginous episode. Among them, 20% do not receive a clear diagnosis. Improved methods, indicators and metrics are necessary to assess the sensory systems related to balance, especially when patients are undergoing treatment for vertiginous episodes. Patients with balance disorders should be monitored for changes at the individual level to gather objective information. In this study, we evaluate the use of the MCQ-Balance (Measure, Classify and Qualify) assessment for examining a patient’s balance progression using tests to measure static balance control and dynamic postural balance with a stabilometric platform.

**Materials and Methods:**

The MCQ-Balance assessment comprises three stages: (i) measuring the progression of each variable between two separate and consecutive days (called sessions) using the Magnitude-Based Decision analysis; (ii) classifying the progression of the patient’s balance with a score; and (iii) qualifying the progression of the patient’s balance from the resulting scores using a set of rules. This method was applied to 42 patients with balance disorders of peripheral or central origin characterised by vertigo as the cardinal symptom. Balance progression was measured using the MCQ-Balance assessment over the course of three months, and these results were compared with the assessment of a clinical expert.

**Results:**

The MCQ-Balance assessment showed an accuracy of 83.4% and a Cohen’s Kappa coefficient of 0.752 compared to the assessment of a clinical expert.

**Conclusion:**

The MCQ-Balance assessment facilitates the monitoring of patient balance and provides objective information that has the potential to improve medical decision making and the adjustment of individual treatment.

## Introduction

Vertigo is an illusion of movement, either of the external world revolving around the individual or of the individual revolving in space (Medical Subject Headings, 2020). It is the cardinal symptom of balance, which leads to a significant reduction in the quality of life and an increase in disability, anxiety and depression (Neuhauser, 2016). There is a high prevalence of balance disorders among elderly people in developed countries (Penger, Strobl & Grill, 2017; Rubenstein & Josephson, 2002). In combination with a gradual increase in the ageing index of the population (Vaupel & Loichinger, 2006; Muir et al., 2013), it has resulted in an increase in the risk of falls of elderly people (Aftab, Robert & Wieber, 2016; Khalaj et al., 2014). Globally, approximately 20 to 30% of the population has a vertiginous episode of various origins and severity over a lifetime (Da Costa Barbosa & Vieira, 2017; Lin et al., 2008; Wolf et al., 1996; Tinetti, 2003). Moreover, 20% of them do not receive a clear diagnosis (Swanenburg et al., 2008).

Vertigo is most often caused by dysfunction resulting from a peripheral or central lesion (Stanton, 2020); therefore, depending on the origin, it can be classified as vertigo of peripheral or central origin (Wipperman, 2014; Baumgartner, 2019; Strupp, Dieterich & Brandt, 2013). Vertigo can also have a cervical origin in cases where the central nervous system is unaffected (except spinal cord syndrome), with a demonstrated relationship between cervicalgia and vertigo (Dieterich & Eckhardt-Henn, 2004; Reiley et al., 2017). Vertigo of cervical origin is identified most frequently in rehabilitation consultations , occurring most often in patients between the ages of 30 and 50 (Jull, 2004; Solomon, 2000).

To analyse the causes of vertigo, the degree of alteration must be measured in isolation or in combination of each balance sensory system (BSS), including the vestibular (VS), visual (ES; eye-sight), and proprioceptive (PS) systems. A vertiginous episode or trauma can affect these systems to a greater or lesser extent, and consequently, the patient’s balance (Hanes & McCollum, 2006; Shumway-Cook et al., 2001). It is therefore necessary to have methods or indicators to determine how the BSS progresses and to standardize the initial evaluation of patients’ balance and its progression, especially during the treatment of a balance disorder (Patrícia Paludette, Fabrício Santana da & Carlos Bolli, 2015).

When it is challenging to establish a clear pathology related to any of the BSSs, or when multiple origins of the condition are found, the clinical diagnosis becomes complicated (Derebery, 2000), and additional measures and tests are required to provide important information to the clinician (Hickey et al., 1990; Martínez Carrasco, 2016; Vellas et al., 1997).

As an alternative or complement to the functional tests, using a stabilometric platform, posturography allows movements of the centre of pressure (COP) in the standing position to be measured. Stabilometric platforms can assess static balance control and dynamic postural balance through different variables and application methods (Ito et al., 2020; Choi & Lee, 2020). It constitutes a functional assessment with medical-legal validity that provides objective information regarding balance disorders in clinical practice (De la Torre et al., 2017; Dounskaia, Peterson & Bruhns, 2018; Lin et al., 2008). Although posturography is a validated assessment, difficulties are encountered with regard to discerning the origin caused by the imbalance pattern. This is because, although sensory analyses suggest a proprioceptive-visual-vestibular pattern, this is not always accurate (El-Kashlan et al., 1998; Stewart et al., 1999; Timothy & Hain, 2019). Related to the above, although the clinical results from traditional posturography are useful, they are insufficient in certain cases, requiring smarter devices (Allum et al., 2002; Di Fabio, 1996).

Posturography devices can provide information on patients’ balance that is useful for clinical decision-making, as a functional assessment value, measuring data related to the patients’ balance; however, in order for such devices to be practical, they must be easy for clinicians to use without consulting external experts (Visser et al., 2008).

Although several balance assessment tests have been applied through a stabilometric platform (Karlsson & Frykberg, 2000), their resulting scores are sometimes complex and difficult to interpret (Peterson et al., 2003). To this end, subjective scoring lacks standardization and can be difficult to interpret, thus making difficult classify the patient balance status, which resulting in difficulties diagnosing balance disorders and identifying the BSS from which the imbalance pattern originates (Jacobs et al., 2006; Visser et al., 2008; Saxena & Prabhakar, 2013).

Posturography reports should involve easily understandable, non-technical language, qualifying the patient’s balance status in an understandable way for clinicians (Von Lubitz & Wickramasinghe, 2006; Visser et al., 2008). Likewise, both validation and standardization of the protocols for reproducibility and a possible comparison with similar studies are required (Visser et al., 2008).

In rehabilitation, it is critical to measure the progression of a patient’s balance between two separate sessions in order to objectively characterize the response to treatments (Hamburg & Collins, 2010); this helps determine whether relevant changes have occurred in the patient at the individual level, thus informing future treatment decision making (Visser et al., 2008; Hopkins, 2017). Regarding this, we can highlight the proposal of (Hopkins, 2017) to assess the change between two measurements in an individual through the magnitude-based decision (MBD) method (Hopkins, 2019), which is used in this work.

The development of the MCQ-Balance assessment method was motivated by these issues, in relation to the necessity of providing objective, easily-interpretable information about patients’ balance that specifies the origin of the pathology. Using a stabilometric platform, this method detects relevant changes between two consecutive balance tests (monitor) in patients with balance disorders, providing objective information about the origin of the imbalance. The MCQ-Balance assessment comprises three separate stages in which the progression of a patient’s balance is measured, then classified, and finally qualified. In this study, the MCQ-Balance assessment was applied to balance disorder patients with vertigo as the cardinal symptom. Subsequently, the results obtained were compared with the evaluation of a specialist clinician.

## Materials & Methods

### Participants

A total of 42 patients with balance disorders characterised by vertigo (of peripheral or central origin) as the cardinal symptom were monitored via balance tests with a stabilometric platform.

The patients were referred by the Primary Care, Otorhinolaryngology and Neurology Services of the Alcañiz Hospital (Teruel, Spain) after being diagnosed with a balance disorder. The methods for determining the deficit, varied according to the service where the diagnosis was made: (i) in Primary Care, medical history was considered; (ii) in Otorhinolaryngology, in addition to the medical history, magnetic resonance imaging, videonystagmography, and tests such as the Dix-Hallpike manoeuvre were used; and (iii) in Neurology, in addition to medical history and magnetic resonance imaging, computerized axial tomography and neurophysiology tests, were used. Table 1 presents the main diagnoses of the patients with respect to peripheral or central deficits. Table 2 shows general and anthropometric characteristics of the patient population. The study included 27 females (64%) and 15 males (36%), and there were no statistically significant differences in any baseline characteristics according to sex (Yin et al., 2009).

From these diagnostic services, patients from the Teruel region (Spain) were referred to the Physical Medicine and Rehabilitation Service (PM&R) of the Alcañiz Hospital, having been identified with vertigo as the cardinal symptom. A doctor (clinician 1) of the PM&R service then evaluated the patients, considering (i) their medical history and physical examination, (ii) previous diagnosis and (iii) the results of the functional balance assessments carried out, such as the Unterberger test (Bartual & Pérez, 1998; Hickey et al., 1990), up and go test (Martínez Carrasco, 2016; Shumway-Cook, Brauer & Woollacott, 2000), and unipodal support test (Vellas et al., 1997).

The selected patients met the following inclusion criteria: (i) between 35 and 70 years old and (ii) having suffered a vertiginous episode of peripheral or central origin in the last year. The following were the exclusion criteria, which were based on (Degani, Leonard & Danna-dos Santos, 2017; Hanes & McCollum, 2006; Swanenburg et al., 2007; Tsukamoto et al., 2015) and were applied in other research (De la Torre et al., 2020a; De la Torre et al., 2020b): (i) presented acute osteomuscular pathology in the lower limbs or lumbar spine, which may alter the outcome of the stabilometric platform, (ii) presented any amputation in the lower limbs, or (iii) presented oncological pathology or was in active treatment with chemotherapy, radiotherapy, or hormonal therapy.

The present study was approved by the Research Ethics Committee of the Community of Aragon (CEICA) (January 16, 2019) and complied the ethical standards of the Declaration of Helsinki (General Assembly of the World Medical Association, 2014).

Prior to the start of the tests, the participants signed a consent form sheet that involved accepting the tests and understanding the purpose of them. The participants in this study has given written informed consent to publish these case details.

**Table 1:** Patients diagnosis according to their deficits.

Peripherical deficit *n* = 32 (76.2%)	Central deficit *n* = 10 (23.8%)
BPPB *n* = 15 (36%)	Ictus *n* = 6 (14%)
Ménière syndrome *n* = 8 (19%)	Neoplasia *n* = 2 (5%)
Vestibular hypofunction *n* = 6 (14%)	Demyelinating disease *n* = 2 (5%)
Otoesclerosis *n* = 3 (7%)	

**Notes.**

BPPV benign paroxysmal peripheral vertigo

**Table 2:** Participant anthropometric characteristics: mean (SD).

Characteristics	Patients (*n* = 42)
Gender (men/women)	15/27
Age (yr)	57.1 (8.7)
Height (cm)	162.8 (7.9)
Weight (kg)	76.5 (15.8)
Body Mass Index (kg/m^2^)	28.7 (5.6)
Foot length (cm)^*^	25.3 (1.1)
Abdominal perimeter (cm)	97.1 (14.9)
Deficit (Peripherical / Central)	32/10

**Notes.**

a Foot length measurements were taken between the proximal and distal points on the foot outline ( Pawar & Dadhich, 2012).

The choice of 42 patients was based on the following formula for sample size calculation involving qualitative variables (Charan & Biswas, 2013):

(1) \begin{eqnarray*}N= \frac{{ \left( {Z}_{1-\alpha /2} \right) }^{2}p(1-p)}{{d}^{2}} \end{eqnarray*}

Here, *Z*_1_ − *α*∕2 is the standard normal variate with *p*_*v*_ < 0.05 (type I error); p is the expected proportion of the research goal in population; and d is the absolute error or precision, as determined by the researcher. We selected a *Z*_1_ − *α*∕2 value of 1.96 (standard, given that py values are considered statistically significant below 0.05), a p-value of 0.7 (based on initial criteria that showed an accuracy of >70%, as shown in the statistical analysis section), and a d-value of 0.15. The resulting N-value was 39.85.

### Instrumentation

**Figure 1:**
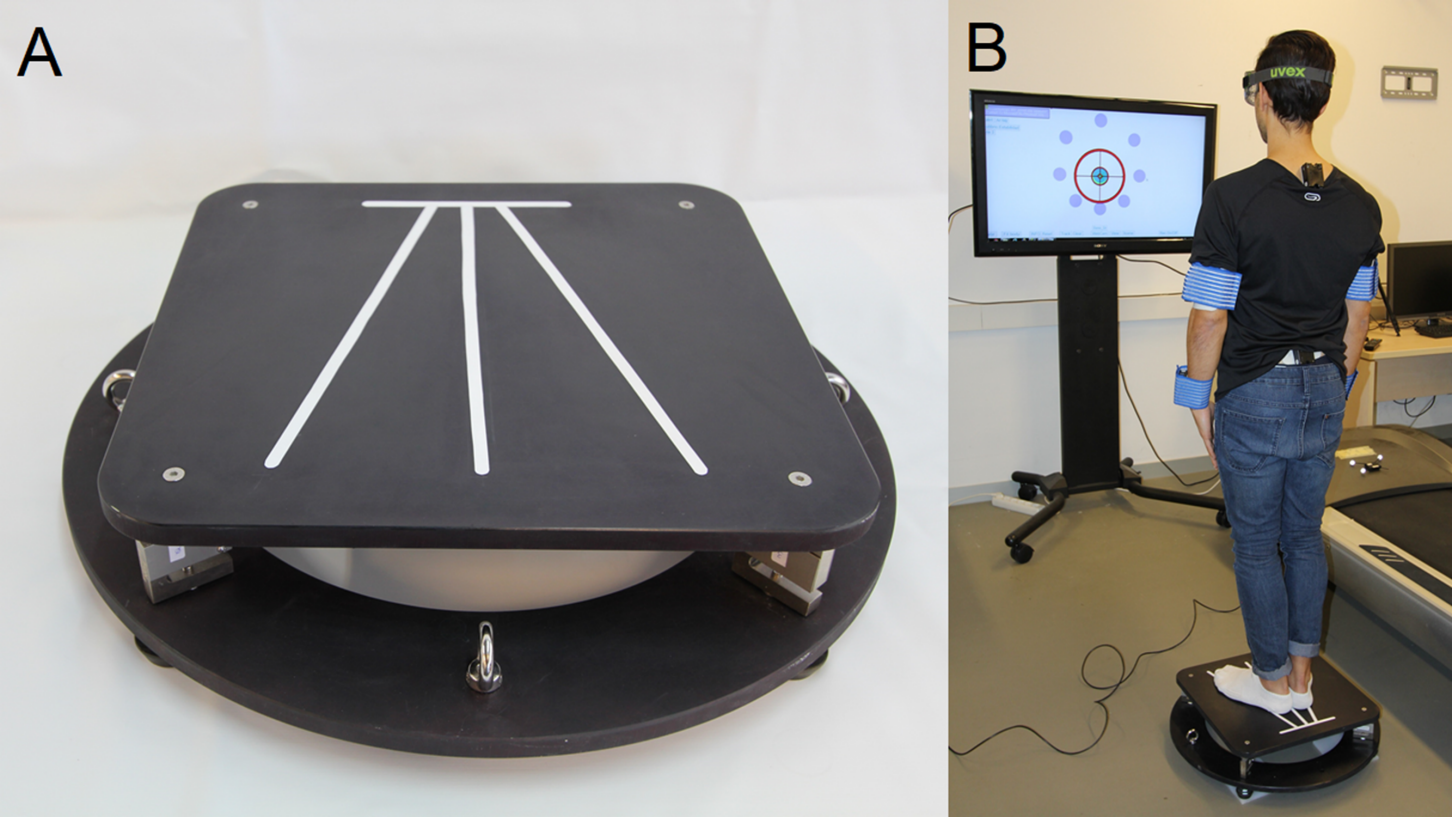
(A) Stabilometric platform and (B) test work environment.

The device used was the stabilometric platform MoveHuman-Dyna UZ, which was designed and manufactured by the IDERGO (Research and Development in Ergonomics, University of Zaragoza, Spain) research group (see Fig. 1). It is a static posturography device designed for research, which comprises four load cells and a lightweight aluminium structure, whose dimensions and characteristics are detailed in the study of De la Torre et al. (2017). The findings of this device can be replicated in a straightforward manner by other researchers, which enhances the applicability of this study. The acquisition and processing of the platform data, as well as the format and method of exporting them, have been carried out according to the procedure used by De la Torre et al. (2017).

Likewise, in accordance with the aforementioned study, the stabilometric platform ‘meets the standards established by the International Society for Posture and Gait Research (ISPGR) for its clinical application’ (Scoppa et al., 2013) in relation to various parameters, such as accuracy, precision, linearity, dimensions, resolution, sampling, and so on. The precision parameters (accuracy, precision, linearity, dimensions and resolution) were obtained through a reliability experiment in which the metrological characteristics of the platform were tested with a gold standard force platform, as well as the error of measurement De la Torre et al. (2017). Processing the force data in function of the cells’ position means we can calculate the real-time position of the trajectory that describes the position of the COP by applying the appropriate formula (López & Calidonio, 2009; Ma et al., 2016). The stabilometric platform has been used in several research projects with patients in different hospitals since 2018, both public (hospital Miguel Servet and university hospital Lozano Blesa (Zaragoza, Spain)) and private (hospital MAZ (Zaragoza, Spain)); all the research projects have been approved by the CEICA Committee. In addition, the characteristics of the platform and its portability make it suitable for clinical use where, for example, the medical office space is limited (Scoppa et al., 2013; De la Torre et al., 2017).

### Protocol

Patients were evaluated by clinician 1 on two different days (sessions) spaced three months apart (first session: pre-session; second session: post-session). After the pre-session, clinician 1 prescribed the rehabilitation treatment according to the specific balance disorder of each patient. Patients with vertigo of peripheral or central origin performed vestibular rehabilitation exercises (Boomsaad, Telian & Patil, 2017). For patients with a specific diagnosis of benign paroxysmal peripheral vertigo (BPPV), the Epley manoeuvre was performed in addition to vestibular rehabilitation exercises (Orejas et al., 2020; Hansson, Persson & Malmström, 2013).

After the evaluation by clinician 1, in each session (pre and post), the patients conducted a set of balance evaluation tests with a stabilometric platform (three months apart between the pre- and post-session). The tests were performed by the PM&R of the Alcañiz Hospital between February and July in 2019. The fieldwork was performed by a team of a clinician (clinician 2), a nurse, and a technician in the same hospital.

The static and dynamic balance were both assessed with a set of tests previously applied in other studies (De la Torre et al., 2020b; De la Torre et al., 2017).

Static balance control was assessed with a test based on the Romberg test and the Modified Clinical Test of Sensory Interaction in Balance (CTSIB-M). In the test patients must maintain their COP within the support base throughout the assessment period - 40 s (De la Torre et al., 2017). Static balance control was assessed in four different conditions, examined consecutively: (1) rigid surface with eyes open (RSEO), (2) rigid surface with eyes closed (RSEC), (3) soft surface with eyes open (SSEO), and (4) soft surface with eyes closed (SSEC).

On the other hand, the dynamic postural balance, which is vital for motor control, was assessed measuring the limits of stability (LOS) that a patient is able to reach and with it, the management capacity of COP (Ku, Abu Osman & Wan Abas, 2016). The inclusion of the LOS, complementary to the assessment of the static balance control, provides additional value to the balance assessment protocol (Lin et al., 2008; Tesio et al., 2013; Salehi et al., 2010).

The specific protocol applied in the tests: the position of the body, arms and feet during the test (De la Torre et al., 2017), environmental conditions (e.g., noise, space, etc.) and the additional instrumentation used as a foam rubber for soft surface or instruments for anthropometric data collection, is the same that (De la Torre et al., 2020a; De la Torre et al., 2020b) used for this stabilometric platform (Fig. 1). This protocol fulfils certain clinical conditions (Swanenburg et al., 2008; Hoving et al., 2005; Benvenuti et al., 1999; Doyle, Newton & Burnett, 2005; it must be fast and should not require multiple repetitions to issue a definite, consistent result (Swanenburg et al., 2008).

At the start of the tests, clinician 2 provided patients with instructions on how to perform the tests, according to similar studies (De la Torre et al., 2020a; De la Torre et al., 2020b). The patients were instructed on how to place their feet on the platform according to the mark placed in the stabilometric platform shown in Fig. 1A. The patients had to be in a standing position, with the arms extended and close to the body. The patients were instructed to keep as still as possible in the static tests; in the LOS, they were instructed to, using only the movement of the ankles without lifting the feet, follow a moving target LOS as explained by De la Torre et al. (2020a). Clinician 2 also provided instructions on how to stimulate abdominal toning, since this has an influence on stability and balance (Ayllón & Fernández, 2006). Patients completed a practice run of each test so that clinician 2 could verify that they understood the procedure, assumed the correct posture, and executed the tests correctly. This also gave the patients the opportunity to get used to the platform and environment, which are considered relevant factors in some balance studies (Taylor et al., 2015).

### Variables

The variables selected for the present study were those determined by De la Torre et al. (2020a). to be more significant in balance assessment studies, which details, and method of obtaining are also explained in the same study. The variables selected for the assessment of the static and dynamic balance were the range of displacement in the anteroposterior and mediolateral directions in mm, area in cm^2^ (surface area covered by the trajectory of the COP), average speed of the COP in mm/s, and RMS position in mm. Additionally, in the LOS test, two more variables were assessed: the COP limits in mm (maximum displacement reached along each axis of the octagon radii), and the “success” variable in percentage (quantification of the management and coordination of the COP along each axis of the octagon radii), both defined in previous studies (De la Torre et al., 2020a; De la Torre et al., 2020b).

### MCQ-Balance assessment method

**Figure 2:**
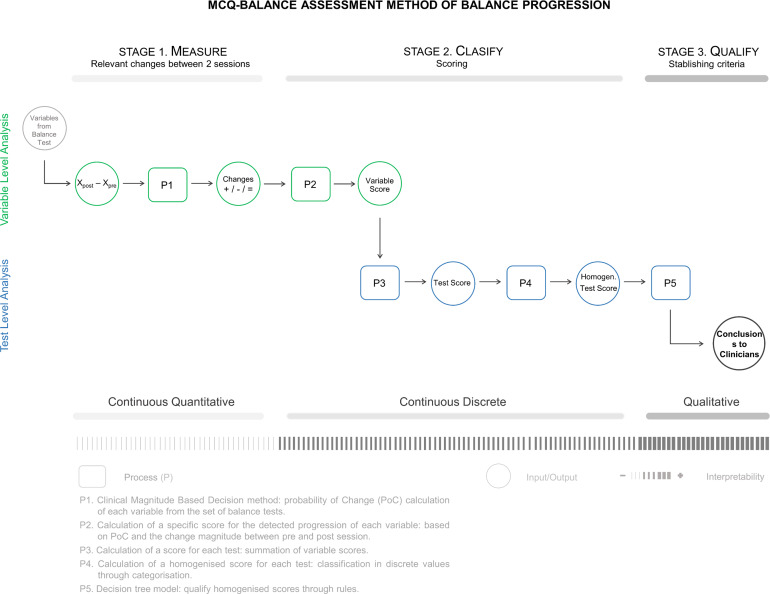
MCQ-Balance assessment method: processes, inputs, outputs from the different stages.

Figure 2 presents the application outline of the MCQ-Balance assessment, which consists of three stages in which the progression of a patient’s balance is Measured (M), Classified (C), and Qualified (Q). The method input is the variables provided by the set of balance tests in two temporal points, that is, the values of the variables in the pre-session and post-session. The variables are analysed individually until stage two, where they are grouped at the test level until the end of the assessment. The application outline shows the inputs and outputs of each stage, as well as the processes (P1-P5) applied to them. It also includes the type of information that is handled and the interpretative changes during the process.

#### Stage 1: Measure

The first stage of the method involves measuring the progression of each variable of the balance tests set by detecting relevant changes between two measures of each variable recorded at different temporal points (e.g., a measure of 26.4 for one session and 27.2 for another session). For this purpose, the process (P1) used in this stage is the statistical method MBD, as described in the Spreadsheet for Monitoring an Individual’s Changes (Hopkins, 2017) (formerly known as magnitude-based inferences) (Hopkins, 2019). According to the MBD method, some inputs are required for each analysed variable:

• Xdif: difference between the measures taken in two temporal points: pre-value (pre-session) and post-value (post-session) (Eq. (1)).
  (2) \begin{eqnarray*}Xdif={X}_{post}-{X}_{pre}\end{eqnarray*}
• MBD threshold: for this method, a threshold (numerical value) must be defined from which a change is considered relevant. In our case, we selected the minimal detectable change (MDC) (Eq. (2)). The implications of this election are explained in the discussion section.
  (3) \begin{eqnarray*}MDC=1.96\sqrt{2}SEM;SEM=SDpool\sqrt{1-ICC}\end{eqnarray*}
  Where the standard deviation (SDpool) is the pooled average between the standard deviation of the test and retest, ICC is the intraclass correlation coefficient (specifically, the calculated coefficient was ICC3, k (similar to ICC2.1) (Ruhe, Fejer & Walker, 2010); the statistical software used for the ICC calculations was the IBM SPSS statistics (IBM Corp, 2017) and the ICC results were classified according to Cicchetti (1994), who provided the following intervals to characterize the ICC inter-rater agreement measures; and SEM is the standard error of measurement. Following the exposed calculation procedure, ICC, SEM and MDC values were obtained in a previous test-retest study (De la Torre et al., 2020a; De la Torre et al., 2020b).
• Short-term typical error (STTE): this represents the error/deviation in the subject’s repeated measurements in a short period for a sample of measurements instead of just one measurement per session, without any substantial change between them (as an intervention, for a long time between measurements, etc.) As proposed by Hopkins (2000) and Hopkins (2017), this input was obtained with a previous short-term reliability study of the balance test set; similar study to the calculation of variables for the MDC (De la Torre et al., 2020a; De la Torre et al., 2020b).

To detect whether the change is relevant between two recorded measures, clinical MBD is followed (Hopkins & Batterham, 2016). This allows us to determine whether the detected progression is positive (beneficial), negative (harmful) or inconclusive.

First, with the value and sign (positive or negative) of Xdif, we determine the tendency of the change towards a positive or negative progression. In the MCQ-Balance assessment method, we follow the following criteria based on (De la Torre et al., 2020a; De la Torre et al., 2020b): for the static balance group, a positive progression is considered if Xdif has a negative sign, and for the dynamic balance group, a positive progression is considered if Xdif has a positive sign.

Subsequently, following the calculation method set forth by Hopkins (2017), the probability of change (PoC in %) is obtained, which can be defined as the probability that the difference between the two values is relevant. This probability corresponds to the percentage of the confidence interval of the difference (calculated using the Xdif and STTE) that is outside of the range (+MDC, -MDC).

Once the PoC is calculated in the method, criteria must be established to consider a positive, negative, or null (unclear) progression of each variable. In a case study following the clinical MBD, a positive PoC that is greater than or equal to 25% corresponds to a relevant positive change, whereas a negative PoC that is greater than or equal to 5% corresponds to a relevant negative change in the patient. In contrast, if the positive PoC is less than 25% or the negative PoC is less than 5%, the change is considered ‘unclear’. The asymmetry between the two intervals is because, in ‘Clinical MBD the effects have an unacceptable risk of harm’ (Hopkins & Batterham, 2016).

#### Stage 2: Classify

The second stage of the method consists of classifying the progression of each patient using a scoring. First, a specific score for each variable is calculated individually. Subsequently, from the scores of each variable, a score is obtained for each test. Finally, the test score is simplified, and a homogenised score (a discrete variable with the values -2, -1, 0, +1 and +2) is calculated for each of them, making it possible to compare the tests with different numbers of variables.

To determine the specific score for each variable (Score.v.mor the score of the variable m), Eq. (3) (P2) was used:

(4) \begin{eqnarray*}Scor{e}_{{v}_{m}}=PoC+CQ\end{eqnarray*}

• PoC: Probability of change for one unit (calculated in 2.4).
• CQ: Quantification of the change that represents the dimensionless difference between the pre- and post-sessions (for one unit) calculated using Eq. (4), in which Xdif is divided by the maximum value of the pre- or post-session. If Xdif is very large (tending to infinity), CQ approaches 1:

(5) \begin{eqnarray*}CQ= \frac{Xdif}{Max(X post;X pre)} \end{eqnarray*}

Considering Eqs. (2) and (3), the range of *Score*_*v*_*m*__ is0 to +2 (positive progression) or -2 to 0 (negative progression). The score per variable is a continuous quantitative variable.

As mentioned above, the present study included five tests (four variants of the Romberg test and the LOS test); therefore, through a calculation based on the variable scores (P3), we obtained five values referred to as Score_Test_n__. In the static balance tests, four situations were considered in which five variables were obtained in each one. In the LOS test, 20 variables were obtained. Equation (5) shows how to calculate the value for Score_Test_n__.

(6) \begin{eqnarray*}Scor{e}_{Tes{t}_{n}}=\sum _{m}^{{N}_{test}}Scor{e}_{{v}_{m}}\end{eqnarray*}

where N_test_ is the number of variables per test. Likewise, in Eqs. (6) and (7), the maximum and minimum scores that the Score_Test_n__ can reach are shown.

(7) \begin{eqnarray*}MaxScor{e}_{Tes{t}_{n}}=2{N}_{test}\end{eqnarray*}

(8) \begin{eqnarray*}MinScor{e}_{Tes{t}_{n}}= \left( -2 \right) {N}_{test}\end{eqnarray*}

For the static balance tests, the maximum and minimum scores were +10 and -10, respectively. For the LOS test, the maximum and minimum scores were +40 and -40, respectively.

Due to the different ranges of scores for each test, it is necessary to perform a classification that homogenises and simplifies the scores independently of the number of variables selected in the previous phases. For this, a process (P4) is conducted in which the global scores are transformed into a discrete quantitative variable through categorisation (González et al., 2020), establishing a classification of five scores between -2 and +2. The proposed intervals are shown in brackets, which were defined based on statistical criteria, the processing and analysis of the data and the view of the clinician 2 involved in the present study:

• -2: high negative progression from Test_n_ (30% *MinScore*_*Test*_*n*__ >*Score*_*Test*_*n*__).
• -1: negative progression from Test_n_ (30% *MinScore*_*Test*_*n*__ ≤*Score*_*Test*_*n*__ <10%*MinScore*_*Test*_*n*__).
• 0: no progression from Test_n_ (10% *MinScore*_*Test*_*n*__ ≤*Score*_*Test*_*n*__ ≤ 10%*MaxScore*_*Test*_*n*__).
• +1: positive progression from Test_n_ (10% *MaxScore*_*Test*_*n*__ <*Score*_*Test*_*n*__ ≤ 30% *MaxScore*_*Test*_*n*__).
• +2: high positive progression from Test_n_ (30% *MaxScore*_*Test*_*n*__ <*Score*_*Test*_*n*__).

#### Stage 3: Qualify

**Figure 3:**
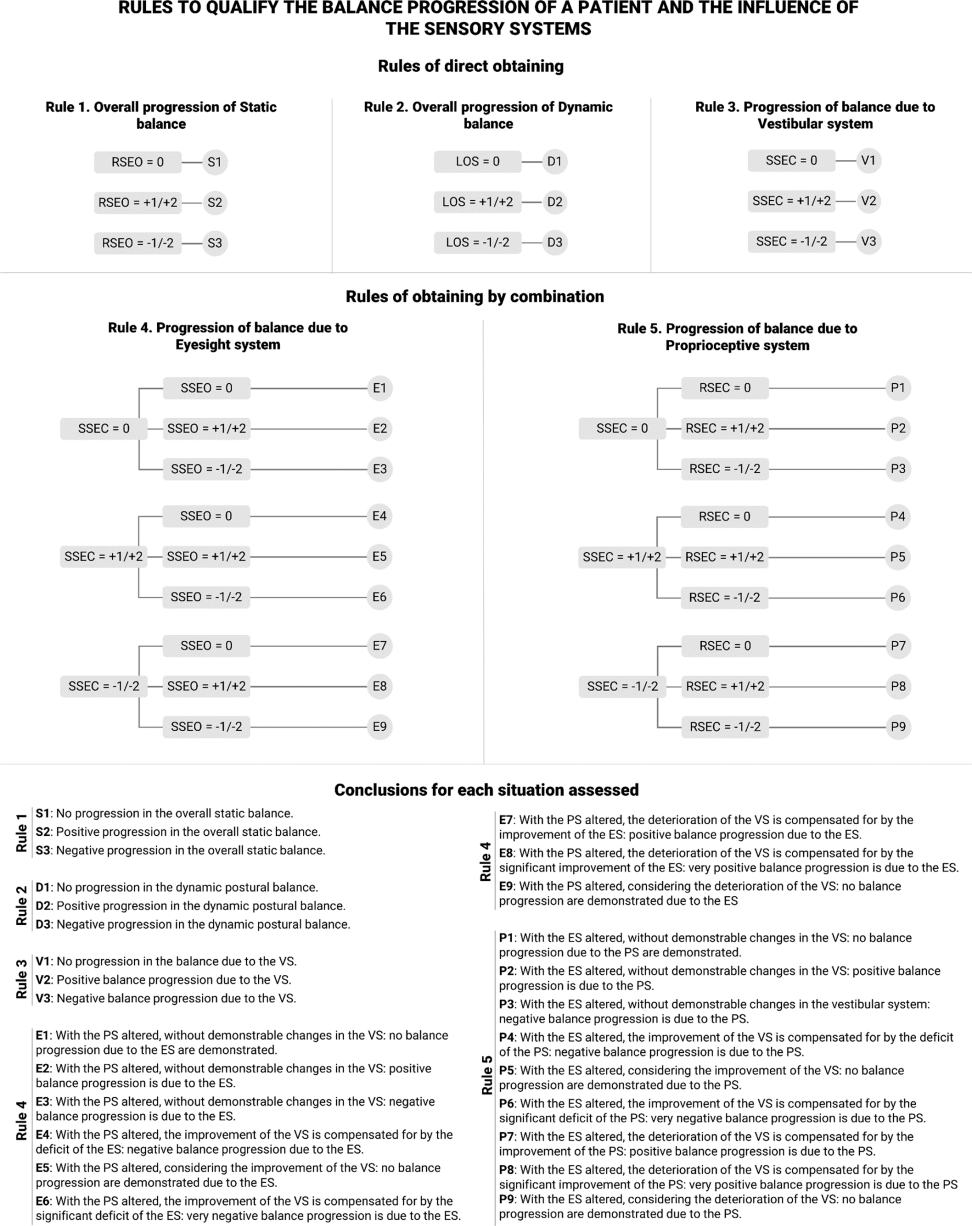
Rules to qualify the balance progression of a patient and the influence of the balance sensory systems. RSEO: Rigid Surface, Eyes Open; RSEC: Rigid Surface, Eyes Closed; SSEO: Soft Surface, Eyes Open; SSEC: Rigid Surface, Eyes Closed. VS: vestibular system; ES: visual system; PS: proprioceptive system. E1–E3: conclusions for the progression of static balance; D1–D3: conclusions for the progression of dynamic postural balance; V1–V3: conclusions for the progression of balance due to VS; S1–S9: conclusions for the progression of balance due to ES; P1–P9: conclusions for the progression of balance due to PS.

The third and final stage involves using established criteria to qualify the progression based on the resulting scores from stage two. For this purpose, rules based on a decision tree model (see Fig. 3) are proposed to qualify the progression of the balance in a patient and the influence of the involved BSS.

As mentioned above, balance is supported by the visual, proprioceptive and vestibular systems. Consequently, in the set of tests presented in Section 2.2, the patient was deprived successively of one or more BSS:

• RSEO: no BSS altered.
• RSEC: ES altered. The balance depends on the VS and PS.
• SSEO: PS altered. The balance depends on the VS and ES.
• SSEC: ES and PS altered. The balance depends only on the VS.
• LOS: no BSS altered. Unique dynamic postural balance test.

Thus, five rules are proposed that lead to their corresponding conclusions (see ‘Conclusions for each situation assessed’ in Fig. 3). The clinicians of the present study developed these conclusions. In addition, the rules are divided into two groups: those directly obtained (1, 2, and 3) and those obtained in combination (4 and 5).

Rules 1 and 2 allow to obtain a global assessment of the progression of the static balance control and the dynamic postural balance of a patient from the RSEO and LOS tests, respectively. Rule 3 allows to obtain an assessment of the influence of the VS on the progression of a patient’s balance, analysing the SSEC test. Rules 4 and 5 assess the influence of the ES and PS, respectively, on the progression of a patient’s balance. These rules result from the combination of SSEC with SSEO (Rule 4) and with RSEC (Rule 5), first analysing the SSEC test and then the corresponding one according to the rule.

### Comparison between the MCQ-Balance assessment and clinician judgment

To analyse the application of the MCQ-Balance assessment, the patient results provided by this method have been compared with the assessment of a clinical expert (clinician 3).

The pre- and post-session data collected by clinician 1 (history and physical examination, diagnosis and functional assessment tests) were assessed by clinician 3 at the end of the field work, which allowed an assessment of the balance progression of each of the 42 patients. To avoid the results being influenced or contaminated by the interaction between the clinicians, there was no contact between them during the research.

The assessment of clinician 3 established three possible categories to evaluate patient progression: positive, null or negative progression (represented by “+”, “=” and “-“, respectively). Regarding the MCQ-Balance assessment, the RSEO variant of the static balance test and LOS test was chosen to make the comparison. This decision was motivated by the fact that, in the RSEO test, the subject has all the BSSs necessary to maintain stability, which corresponds to the standard situation where all BSSs are intact; it is a more favourable test and more consistent with the performance of daily living activities. In addition, in the LOS test (where the capacity or stability limits of patients are measured), the patient is also not deprived of any BSS; therefore, both tests are performed under the same conditions, which we consider in favour of the assessment used in this study (between the results of the pre-treatment and post-treatment session).

Likewise, and since clinician 3 could only establish a classification in three categories, the MCQ-Balance assessment scores have been simplified to a positive (+2 and +1 simplified to ‘+’), null (0 simplified to ‘=’) and negative (-2 and -1 simplified to ‘–’) progression in order to properly conduct the comparison.

### Statistical analysis

We used the statistical software IBM SPSS statistics Version 25 (IBM Corp, 2017) for the statistical analysis of the data. To make the comparison between the MCQ-Balance assessment results and the assessment of clinician 3, the Cohen’s Kappa statistical coefficient (*κ*) was chosen (Cantor, 1996), which is used to measure inter-rater reliability for qualitative (categorical) items. Likewise, the confusion matrix was calculated to obtain the accuracy and percentage of false negatives.

Regarding the results of the comparison, it would be reasonable to obtain a Cohen’s Kappa coefficient of a moderate or higher category (index above 0.4), as well as an accuracy of more than 70% to minimize the number of false negatives.

## Results

The results of the statistical analysis of the comparison between the MCQ-Balance assessment and the evaluation of clinician 3 are presented below.

### Stage 1

Regarding phase 1, the average PoC is presented for each patient’s tests (see Table 3). The motivation for the choice of PoC is the main output of phase 1 and, therefore, the most representative variable. Due to the volume of information handled, it was not possible to include the information at the variable level as explained in the method; however, the information of each variable from the pre- and post-sessions (pre-value, post-value, difference, MDC, STTE, PoC, CQ and the scores of each variable) of the patients’ tests has been calculated and compiled as supplementary material.

### Stage 2

The results related to stage 2 correspond to the homogenised scores of the five tests of the 42 patients, as presented in Table 4. This score is a discrete value between -2 and +2; negative values (-2 and -1) indicate negative progression, null values (0) indicate no progression and positive values (1 and 2) indicate positive progression.

**Table 3:** Stage 1 results: Probability of change of each patient and test.

ID	Def	RSEO	RSEC	SSEO	SSEC	LOS	ID	Def	RSEO	RSEC	SSEO	SSEC	LOS
01	P	0.12	0.79	0.16	0.40	0.11	22	P	0.22	0.29	0.26	0.70	0.72
02	P	−0.06	−0.12	−0.17	−0.38	−0.08	23	P	0.58	0.02	0.06	0.35	0.16
03	P	0.19	−0.03	0.15	−0.05	0.09	24	C	0.09	0.77	0.69	−0.74	0.01
04	P	−0.21	−0.80	−1.00	−0.52	−0.23	25	P	−0.30	−0.40	−0.60	−0.37	0.05
05	P	−0.49	−0.49	−0.30	0.06	−0.10	26	C	0.21	−0.06	−0.06	0.22	0.19
06	P	−0.14	−0.26	0.02	−0.62	−0.14	27	P	−0.15	−0.39	0.48	−0.31	−0.15
07	P	0.39	−0.02	0.46	0.18	0.34	28	P	0.00	−0.19	−0.03	0.35	−0.07
08	P	0.13	−0.31	0.21	−0.22	0.15	29	P	0.18	1.00	0.58	0.23	−0.07
09	P	−0.66	−0.02	0.15	−0.46	0.13	30	C	0.62	0.40	0.76	0.26	−0.21
10	P	−0.57	−0.02	0.15	−0.93	0.15	31	P	0.05	−0.15	−0.34	−0.13	0.06
11	P	−0.05	−0.05	−0.03	0.04	−0.07	32	P	−0.78	0.09	−0.23	−0.94	−0.32
12	P	−1.00	n/a	n/a	n/a	n/a	33	P	−0.04	−0.73	0.19	−0.06	0.00
13	P	0.25	−0.06	−0.39	−0.52	0.16	34	C	−0.80	−0.69	0.28	n/a	n/a
14	C	−1.00	−0.98	n/a	n/a	n/a	35	P	0.23	−0.19	0.26	0.18	0.17
15	P	−0.14	−0.64	−0.90	0.98	−0.05	36	P	0.10	−0.43	0.22	−0.14	−0.16
16	P	0.19	0.94	0.59	0.99	0.16	37	P	0.13	1.00	−0.03	0.29	0.16
17	C	0.17	0.00	−0.07	0.75	0.43	38	C	−0.86	−0.32	0.07	−0.71	0.00
18	P	−0.07	−0.30	0.84	−0.26	0.13	39	P	0.15	0.55	−0.06	−0.08	0.17
19	P	−0.40	−0.25	0.50	0.04	−0.26	40	C	−1.00	n/a	n/a	n/a	n/a
20	C	−0.20	−0.22	−0.64	−0.43	0.58	41	C	−0.68	−0.63	−0.84	n/a	n/a
21	P	0.07	0.05	−0.38	−0.08	0.11	42	P	−0.19	−0.11	−0.11	−0.03	0.01

**Notes.**

ID patient identifier Def vertigo deficit P peripherical deficit C central deficit n/a test not performed RSEO rigid surface eyes open RSEC rigid surface eyes closed SSEO soft surface eyes open SSEC soft surface eyes closed LOS limits of stability

### Stage 3

Qualification of the scores of each patient, a process conducted in stage 3, is presented in Table 4 with the same identifying code detailed in Fig. 3, where the conclusions are presented based on the scores obtained.

### Comparison between the MCQ-Balance assessment and clinician judgment

The results of the comparison between the MCQ-Balance assessment and the assessment of clinician 3 for the RSEO and LOS tests are presented in Table 5 and 6, respectively. They include the confusion matrix, Cohen’s Kappa coefficient with its significance (p-value) and the number of false negatives.

As shown in Table 6, for the RSEO test, Cohen’s Kappa coefficient is 0.752 (between 0.61–0.80 as substantial (McHugh, 2012), the accuracy is 83.4% between the two assessments and there are no false negatives.

As shown in Table 6, for the LOS test, Cohen’s Kappa coefficient is 0.581 (between 0.41–0.60 as moderate (McHugh, 2012)), the accuracy is 72.9% between the two assessments and there are four false negatives, including three cases where the method did not detect changes and the clinical expert estimated worsening as well as one case where the method detected positive progression and the clinical expert estimated worsening.

## Discussion

In this study, the MCQ-Balance assessment showed an accuracy of 83.4% compared to evaluation by an expert clinician for the detection of relevant changes in balance in patients with balance disorders. The methodology used in this study is easily reproducible, given the wide availability of the resources used.

Few studies have focused on the clinical utility of posturography at the individual patient level (Visser et al., 2008). Likewise, although posturography is considered the gold standard, limitations exist regarding its use as a functional assessment (Climent Barbera, 2003). Thus, MCQ-Balance assessment method proposed, focuses on the individualised monitoring of patients, try to respond to this problem. Indeed, the transformation of information from continuous quantitative variables to conclusions in medical language facilitates the clinical interpretation of the results, providing greater intelligence to posturography devices (which is a limitation detected in posturography reports) (Climent Barbera, 2003).

**Table 4:** Stage 2 results—homogenised scores—and stage 3 results—conclusions—of each patient.

			STAGE 2: CLASIFY					STAGE 3: QUALIFY				
ID	**VO**	**CA**	**RSEO**	**RSEC**	**SSEO**	**SSEC**	**LOS**	**R1**	**R2**	**R3**	**R4**	**R5**
01	P	=	0	2	0	1	0	S1	D1	V2	E4	P5
02	P	=	0	0	−1	−1	0	S1	D1	V3	E9	P7
03	P	+	1	0	0	0	1	S2	D2	V1	E1	P1
04	P	–	−1	−2	−2	−2	−1	S3	D3	V3	E9	P9
05	P	–	−2	−2	−1	0	−1	S3	D3	V1	E3	P3
06	P	=	−1	−1	0	−2	0	S3	D1	V3	E7	P9
07	P	+	2	0	2	1	1	S2	D2	V2	E5	P4
08	P	=	0	−2	1	−1	0	S1	D1	V3	E8	P9
09	P	+	−2	0	1	−1	1	S3	D2	V3	E8	P7
10	P	–	−2	0	1	−2	0	S3	D1	V3	E8	P7
11	P	+	0	0	0	0	0	S1	D1	V1	E1	P1
12	P	–	−2	n/a	n/a	n/a	n/a	S3	n/a	n/a	n/a	n/a
13	P	+	1	0	−1	−2	1	S2	D2	V3	E9	P7
14	C	–	−2	−2	n/a	n/a	n/a	S3	n/a	n/a	n/a	n/a
15	P	+	−1	−2	−2	2	0	S3	D1	V2	E6	P6
16	P	+	1	2	2	2	1	S2	D2	V2	E5	P5
17	C	+	1	0	0	2	1	S2	D2	V2	E4	P4
18	P	=	0	−1	2	−1	0	S1	D1	V3	E8	P9
19	P	=	−2	−1	2	0	−1	S3	D3	V1	E2	P3
20	C	–	−1	−1	−2	−1	2	S3	D2	V3	E9	P9
21	P	=	0	0	−1	0	0	S1	D1	V1	E3	P1
22	P	+	1	1	1	2	2	S2	D2	V2	E5	P5
23	P	+	2	0	0	1	1	S2	D2	V2	E4	P4
24	C	=	0	2	2	−2	0	S1	D1	V3	E8	P8
25	P	–	−1	−2	−2	−1	0	S3	D1	V3	E9	P9
26	C	+	1	0	0	1	1	S2	D2	V2	E4	P4
27	P	–	−1	−1	2	−1	−1	S3	D3	V3	E8	P9
28	P	=	0	−1	0	1	0	S1	D1	V2	E4	P6
29	P	+	1	2	2	1	0	S2	D1	V2	E5	P5
30	C	+	2	1	2	1	−1	S2	D3	V2	E5	P5
31	P	=	0	0	−1	0	0	S1	D1	V1	E3	P1
32	P	–	−2	0	−1	−2	−1	S3	D3	V3	E9	P7
33	P	=	0	−2	1	0	0	S1	D1	V1	E2	P3
34	C	–	−2	−2	1	n/a	n/a	S3	n/a	n/a	n/a	n/a
35	P	+	1	−1	1	1	1	S2	D2	V2	E5	P6
36	P	=	0	−2	1	−1	−1	S1	D3	V3	E8	P9
37	P	+	1	2	0	1	1	S2	D2	V2	E4	P5
38	C	–	−2	−1	0	−2	0	S3	D1	V3	E7	P9
39	P	+	1	2	0	0	0	S2	D1	V1	E1	P2
40	C	–	−2	n/a	n/a	n/a	n/a	S3	n/a	n/a	n/a	n/a
41	C	=	−2	−2	−2	n/a	n/a	S3	n/a	n/a	n/a	n/a
42	P	+	−1	0	0	0	0	S3	D1	V1	E1	P1

**Notes.**

ID patient identifier Def vertigo deficit P peripherical deficit C central deficit n/a test not performed RSEO rigid surface eyes open RSEC rigid surface eyes closed SSEO soft surface eyes open SSEC soft surface eyes closed LOS limits of stability R1...R5 Rules from stage 3, consult figure 3 S, D, V, P, E consult conclusions from figure 3

Stages two and three of the method are adapted to clinical needs because they are the result of multidisciplinary work involving clinicians and technicians. This highlights the relevance of the conclusions that the MCQ-Balance method can generate from the results of the balance tests, which have been defined and written by the clinicians involved in the present study. Likewise, the definitions of the intervals of the homogenised scores have been adjusted according to the patients that have been assessed by the clinician 2.

The proposed method has advantages over traditional posturography; however, it is necessary to discuss certain issues and decisions related to the application process, which are explained below.

The first consideration refers to the chosen MBD threshold, a numerical value from which a change is considered relevant. Regarding this, the MDC has been selected as the reference value in the present study because it represents the random balance variability in addition to the measurement errors of the device and the experiment (Furlan & Sterr, 2018; Steffen & Seney, 2008). We choose the MDC, rather than the minimal important difference (MID), as the MBD threshold (De Vet & Terwee, 2010), consistent with previous studies (De la Torre et al., 2020a; De la Torre et al., 2020b).

**Table 5:** MCQ-Balance assessment and clinician judgment comparative: rigid surface eyes open test.

			MCQ-Balance Assessment			Total
			**–**	**=**	**+**	
Clinical Expert Assessment	**–**	**N**	**12**	0	0	12
		**%**	**28.6%**	0%	0%	28.6%
	**=**	**N**	3	**10**	0	13
		**%**	7.1%	**23.8%**	0%	31%
	**+**	**N**	3	1	**13**	17
		**%**	7.1%	2.4%	**31%**	40.5%
Total		**N**	18	11	13	**42**
		**%**	42.9%	26.2%	31%	**100%**
Symmetric measure	**Kappa**	**0.752**	*P*-Value	0.000	**False Negatives**	**00%**

**Notes.**

N count of each case % percentage of total

**Table 6:** MCQ-Balance assessment and clinician judgment comparative: limits of stability test.

			MCQ-Balance Assessment			Total
			**–**	**=**	**+**	
Clinical Expert Assessment	**–**	**N**	**4**	3	1	8
		**%**	**10.8%**	8.1%	2.7%	21.6%
	**=**	**N**	2	**10**	0	13
		**%**	5.4%	**27%**	0%	32.4%
	**+**	**N**	1	3	**13**	17
		**%**	2.7%	8.1%	**35.1%**	45.9%
Total		**N**	7	16	14	**37**
		**%**	18.9%	43.2%	37.8%	**100%**
Symmetric Measure	**Kappa**	**0.581**	*P*-Value	0.000	**False Negatives**	**410.8%**

**Notes.**

N count of each case % percentage of total

The scoring proposed in the present work makes it possible to simplify the interpretation of the results of balance monitoring at the patient level. For this, the scoring allows the results to be standardized to enable a comparison between tests of the same patient and even between studies of different patients.

In the present work, and according to De la Torre et al. (2017), the considered variables have the same importance and are assigned the same weight. However, future studies might advise assigning a different weight to each variable depending on its importance in improving the sensitivity of the MCQ-Balance method for diagnostic purposes. In this case, the maximum and minimum achievable score for each test would be based on the weights assigned to each variable.

The choice of the five intervals to establish the homogenised scores was medically motivated. Clinically, it makes sense to make a five-level classification because the progression of the patient is towards improvement, maintenance, or deterioration of the patient’s clinical picture (Porta, 2014), assessing the existing graduation in improvement or deterioration. The multidisciplinary agreement reached in the present work combined with the experience of fieldwork and data processing has been concluded at the presented intervals.

Regarding the conclusions in medical language resulting from the method, the ability to portray the influence of the three BSS involved in balance is highlighted in the progression of a patient’s balance. In this way, the method facilitates the clinician to adapt medical treatment, focusing on the balance disorder of the patient.

MCQ-Balance assessment exceeded 70% accuracy (relative to the assessment of clinician 3) for both the RSEO test and the LOS test, and its Cohen’s Kappa coefficient was >0.4. Therefore, the MCQ-Balance assessment met the accuracy goals we initially established. However, the differences between the two comparisons should be highlighted. While there were no false negatives in the comparison with the RSEO test, with the LOS test, there were four (10.8% of the sample). This is explained by the possible learning factor associated with this test (Wrisley, 2007), although 4 of the 37 patients who completed this test is not a representative sample; similar to the comparison with RSEO, there are more cases in which the method determined a negative progression (worsening) where clinician 3 did not. This may be due to the increased sensitivity of the method when detecting worsening that is not visible to the clinician with traditional assessment tools. Finally, we would like to establish that the decision to choose these two tests has been motivated because all BSSs are intact, a situation more in line with the performance of daily living activities. In our opinion is the best adaptation to the assessment of the clinician 3. Although we consider the reliability obtained in this study adequate (>70%), delving into this type of comparison could result in further improved accuracy.

The simplicity of the MCQ-Balance assessment, as well as its portability and reproducibility, make it possible to systematize its use in the clinic as a complementary evaluation tool. However, future research should focus on verifying the viability of continued clinical use of this assessment, as well as its incorporation into the dynamics of a hospital rehabilitation service.

The influence of participant characteristics has not been analysed because there is no significant difference (gender) and it is not within the scope of the research; however, it was observed that older patients showed less positive progression relative to younger patients. The analysis of the possible influences of the anthropometric variables will be addressed in a future study.

Regarding the progression of the patients, it can be observed that there is no trend in improvement (positive progression) of the sample. The main reason lies in the nature of the prescribed treatments. To achieve effectiveness in rehabilitative treatment, patients need to be constant in performing the prescribed treatment, which is a great handicap of rehabilitation (regardless of subspecialty) (Tapias, 2014; Essery et al., 2017). Likewise, some cases of fear in the patients were detected in the post-session due to a negative experience in the pre-session. This explains certain cases that present a negative progression provided by the method. This problem is frequent in studies of balance disorders (Visser et al., 2008; Timothy & Hain, 2019). However, we tried to minimise the problem with additional safety measures, such as the presence of the clinician 2 and a nurse around the patient during the tests.

The lack of portability of current posturography devices is problematic. More portable devices would reduce costs (given the quicker installation process and smaller space requirements) and allow the sharing of devices between different medical centres. However, the high price of more portable devices limits their accessibility and applicability (Uebbing, 2016). The reduced cost of the device used in this study, as well as its portability, supports use in lower income countries that may be unable to invest in high-cost posturography equipment.

We acknowledge the major limitation inherent to the applied treatments, although the purpose of the study was not to assess the efficacy of treatments for balance disorders. Likewise, in the assessment of those patients diagnosed with BPPV to whom the Epley manoeuvre was applied, no greater positive progression was detected than the rest of the sample due to the use of a specific treatment. The effectiveness of the treatments will be addressed in a subsequent study with a sample similar to that of the present study. Likewise, future studies should compare the MCQ-Balance assessment with the BESTest (Padgett, Jacobs & Kasser, 2012). Besides, new output measures should be added, such as the sway directional index, sway vector (Błaszczyk, 2016), or even fractal dimension (Błaszczyk & Klonowski, 2001); as well as introduce cognitive tasks (Raymakers, Samson & Verhaar, 2005); De la Torre, Bonnet et al., 2020). Finally, future studies should investigate the possibility of further improving the accuracy MCQ-Balance assessment by incorporating machine learning techniques.

Regarding the implications and possibilities of the assessment method MCQ-Balance, note that it is extrapolated to other cases of balance assessment with different tests, variables, and perspectives (e.g., balance during gait or by combining the test with cognitive tasks). Therefore, the conclusions transcend the present study.

## Conclusions

This study assessed the accuracy and clinical utility of the MCQ-Balance assessment for measuring balance progression in patients with balance disorders. The results obtained with the MCQ-Balance assessment showed remarkable similarity to the assessment of an expert clinician, demonstrating the validity of this new method. We conclude that the proposed method provides objective information that facilitates the monitoring of patients with balance disorders and measurement of alterations in BSS.

##  Supplemental Information

10.7717/peerj.10916/supp-1 Supplemental Information 1 Patients’ raw data.

## Abbreviations

BPPV: Benign Paroxysmal Peripheral Vertigo
BSS: Balance Sensory System
CEICA: Research Ethics Committee of the Community of Aragon
COP: Centre Of Pressure
CQ: Quantification of the Change
CTSIB-M: Modified Clinical Test of Sensory Interaction in Balance
ES: Eye-Sight System
ICC: Intraclass Correlation Coefficient
IDERGO: Research and Development in Ergonomics
ISPGR: International Society for Posture and Gait Research
*κ*: Cohen’s Kappa Statistical Coefficient
LOS: Limits of Stability
MBD: Magnitude-Based Decision
MCQ: Measure, Classify and Qualify
MDC: Minimal Detectable Change
MID: Minimal Important Difference
PM&R: Physical Medicine and Rehabilitation Service
PoC: Probability of Change
PS: Proprioceptive System
RSEC: Rigid Surface with Eyes Closed
RSEO: Rigid Surface with Eyes Open
SDpool: Pooled Average between the Standard Deviation of the Test and Retest
SEM: Standard Error of Measurement
SSEC: Soft Surface with Eyes Closed
SSEO: Soft Surface with Eyes Open
STTE: Short-Term Typical Error
VS: Vestibular System
Xdif: Difference Between the measures taken in two Temporal Points

## Additional Information and Declarations

### Data Availability

The following information was supplied regarding data availability: Raw data are available in as a Supplemental File.
